# Entropy Measures Can Add Novel Information to Reveal How Runners' Heart Rate and Speed Are Regulated by Different Environments

**DOI:** 10.3389/fpsyg.2019.01278

**Published:** 2019-06-04

**Authors:** Juliana Exel, Nuno Mateus, Bruno Gonçalves, Catarina Abrantes, Julio Calleja-González, Jaime Sampaio

**Affiliations:** ^1^Creative Lab Research Community, Research Center in Sports Sciences, Health Sciences and Human Development, CIDESD, University of Trás-os-Montes and Alto Douro, Vila Real, Portugal; ^2^Geron Research Community, Research Center in Sports Sciences, Health Sciences and Human Development, CIDESD, University of Trás-os-Montes and Alto Douro, Vila Real, Portugal; ^3^Department of Physical Education and Sports, University of the Basque Country (UPV-EHU), Bilbao, Spain

**Keywords:** heart rate, speed, running, training, entropies, dynamical systems

## Abstract

Ecological psychology suggests performer-environment relationship is the appropriate scale for examining the relationship between perception, action and cognition. Developing performance requires variation in practice in order to design the attractor-fluctuation landscape. The present study aimed to identify the effects of varying levels of familiarity and sensorimotor stimuli within the environment in runners' speed and heart rate (HR) regularity degree, and short-term memory Twelve amateur runners accomplished three 45-min running trials in their usual route, in an unusual route, and an athletics 400-m track, wearing a GPS and an HR monitor. Sample entropy (SampEn) and complexity index (CI), over speed and HR, were calculated. Pre and post-trial, participants performed the Backward Digit Span task for cognitive assessment. Higher entropies were found for the 400-m track, compared to the usual and unusual routes. Usual routes increased speed SampEn (63% of chances), but decreased HR CI when compared to unusual routes (60% of chances). Runners showed higher overall short-term memory performance after unusual routes, when compared to usual routes (85% of chances), indicating positive relation to attentional control. The contexts of practice may contribute to change predictability from single to multiple timescales. Thus, by considering that time structuring issues can help diagnosing habituation of training routes, this study brings novel information to the long-term process of training.

## Introduction

Ecological psychology suggests that the association between the performer of an action and the practice environment is the most appropriate scale of analysis for examining the relationship between perception, action, and cognition. Related to sports, running is one of the most practiced activities worldwide (Hulteen et al., 2017) and its popularity is reflected in the continuous increase in the number of master athletes (>40 years old) participating in endurance and ultra-endurance events over the past years (Zaryski and Smith, 2005; Lepers et al., 2016). Middle- and long-distance runners perform a considerable part of training outdoors, which has been positively associated with psych-physiological markers along with peoples' well-being and health status (Triguero-Mas et al., 2017). However, the tendency to repeat and stabilize decisions and behaviors in daily life (Betsch, 2011), also reflected in the choices of training environments, might negatively influence the interactions among the complex network of physiological pathways existing on the human body. The reduction in such interactions may lead to reduced point-to-point fluctuations in the outcomes (Lipsitz, 2002), leading to an unwanted decreased capacity of the body in adapting to the important effects promoted by physical activity on health and performance (O'Donovan et al., 2010).

Thus, developing performance requires variation in the training stimuli in order to challenge the attractor-fluctuation landscape and offset the internal and external perturbations that act on the body during exercise, so body system adaptive capacity can be increased (van Emmerik and van Wegen, 2002). Ecological-based research demonstrates that athletes regulate exercise intensity according to the environment (Konings and Hettinga, 2017), number and behavior of eventual opponents (Gonçalves et al., 2017), and stage of competition (Hettinga et al., 2017), by controlling speed in the actualization of the available affordances. Literature encourages the introduction of challenging motor demands to increase the adaptability of body complex motor patterns, which is key to improving sport performance in various environments (Button et al., 2006; Moras et al., 2018). From this perspective, it seems that information about the task might not be only stored in the brain, but also granted by the local environment and over time (Schmidt, 2007; Gibson, 2014). Additionally, variability is one of the presumable links between cognition and action, which is a current hot topic in sports sciences and health. Constant, but variate, external stimulation induces positive and enduring modifications in adults' brain structure and functions by maintaining the working plasticity properties, thus generating major positive impacts in terms of lower-level body functions (Woollett and Maguire, 2011). However, the relationship between the regulation of action and the performance determinants in sports has not been totally explained. In fact, it is still not clear how habituation over contexts (i.e., training in the same place or in the same route) or environments (i.e., training with variate or repetitive landscape) of practice is reflected in biological complexity and cognition, which would provide evidence on to what extent basic modifications in practice benefits performance and health.

In this sense, the dynamical systems perspective has presented a valued approach to assessing changes in variability, providing complementary information of the underlying dynamics of biological signals under various conditions (Costa et al., 2002). Using non-linear methods for quantifying the level of unpredictability at all relevant time-resolution levels of performance outcomes, as sample and multiscale entropies, provides measures of its complexity. For example, heart rate activity (HR) is a key internal variable that provides information about the cardiovascular responses to exercise and sports (Hautala et al., 2003; Kiviniemi et al., 2010). The *beat-to-beat interval* (R-R interval) and *beats-per-minute* (BPM) are the standard heart beat measurements, and differ in the degree to which they provide information about the heart beat dynamics (Wallot et al., 2013). BPM is assumed as the standard statistic for exercise prescription and, therefore, is the most common available measure. Although R-R intervals preserve the natural variability of HR activity, some studies might critically depend on the retained temporal structure of HR time-series, which are characteristic of BPM, but not R-R interval (Konvalinka et al., 2011).

Constant, but variate, external stimulation induces positive and enduring modifications in adults' brain structure and functions by maintaining its plasticity properties working, thus generating major positive impacts in terms of lower-level body functions. Therefore, the present study aimed to quantify the relationship between variate environmental features to the degree of regularity in cognition, and mechanical and physiological variables. The current research protocol was designed to use training routes with different degrees of familiarity and sensorimotor stimuli to understand how variability is associated to motor and cognitive function. This study hypothesized that routes frequently used by runners (usual routes) yield higher regularity in biological signals when compared to new routes (unusual routes).

## Materials and Methods

### Participants

The sample comprised 12 middle and long-distance runners (42 ± 7.1 years, 174.3 ± 8 cm height, and 73.1 ± 10.1 kg body mass). To be included in the study, they should be engaged in amateur competition and an organized training schedule including specific outdoor running practices for, at least, 3 times a week. They also would have to present no history of musculoskeletal, neurological, or orthopedic disorder for the last year. Participants were fully informed about the purpose, benefits and risks of the study, and provided written informed consent before the study started. The study protocol was approved and followed the guidelines stated by the Ethics Committee of the of University of Trás-os-Montes and Alto Douro, based at Vila Real (Portugal), and conformed to the recommendations of the Declaration of Helsinki.

### Protocol

The participants were asked to accomplish three 45-min running trials in 3 different scenarios: their usual training route, an unusual route and at a standard 400-m track. The usual route would have to be the most practiced or the standard trail/road route covered at regular training sessions. For the unusual route, each participant would have to choose a trail/road route which had never been chosen for training before the assessments. The 400-m track was chosen to represent an environment with a flat, repetitive, and monotonous sensory-motor stimuli of practice. Participants were asked to perform the running trials during their usual training days.

Running speed and heart rate BPM were assessed using GPS (SPI-PRO, GPSports, Canberra, ACT, Australia) synchronized with a HR belt (Polar Team Sports System®, Polar Electro Oy, Finland), at 5 Hz sampling. The course altitudes for all training trials were obtained using the latitude and longitude data from the GPS Visualizer's Elevation Lookup Utility (Schneider, 2013).

Experiments have demonstrated that there is a difference in the interactions people experience during tasks performed in different environments, reflected in the cognitive functioning at the short-term memory level (Berman et al., 2008). To assess short-term memory, we used the Backward Digit Span task, which measures short-term memory in adults through directed-attention mechanisms, as items are moved in and out of the focus of attention during tasks such as running outdoors (St Clair-Thompson, 2010). The test was adapted into a dedicated game app for mobiles that was installed in the personal smartphone of all participants. When started, the game would show a sequence of numbers on the screen. Then, a blank space and keyboard would appear on the screen to allow the user to type the sequence of numbers, but in the reverse order. The game included different number sequences, so the user was challenged across levels. In each level, an extra number was added to the sequence. The game was interrupted when 2 trials were missed in the current level. The final score of the game was available to the user in the end, including the option of being sent to an e-mail address dedicated to the study. The game final score accounted for the last level achieved, the number of correct answers, number of failed answers, and average time per answer.

Participants followed the same protocol for all running trials. Before each trial, the runners would have to turn on and wear the GPS and HR monitor, and then perform the backward digit span test. They would have to email the final scores of the test right after finishing the game. After the running trials, participants were instructed to perform the short-memory test again, email the results, to then undress and turn off the tracking device.

### Data Processing

Data analyses were performed using the middle 20 min of each running trial. Speed data was smoothed using LOESS quadratic fit function (Cleveland, 1979) with 0.001 as the smoothing parameter, which is related to the size of the window used for each partial adjustment. This smoothing parameter was defined after observing the quality of time series derivatives and residual analysis (Winter, 2009).

The presence of non-linear features in the data was identified by estimating the difference between the sample entropy (SampEn) calculated for both time series and its surrogates, as well as analyzing the highest Lyapunov Exponent. The amplitude-adjusted Fourier transform algorithm was used for the HR time series, and pseudo-periodic surrogate function was applied to the speed time series (Stergiou, 2016). Low levels of regularity in the HR time series were found, so a 3rd order polynomial was fitted to de-trend the data (Stergiou, 2016). SampEn is a method of modified entropy computation from the approximate entropy method and consists of four steps: reconstruction, definition of distance, definition of the criterion for similarity, and entropy calculation (Lee and Choi, 2018).

In the first step, for a *N* points time series *x*_*N*_ = {*x*_1_, *x*_2_, …, *x*_*n*_}, the reconstruction of *x*_*N*_ into multidimensional vectors is performed as follows:

(1) $$X_{m}^{\partial }\left(i\right)=\left\{x_{i},\;x_{i+\partial },\;\ldots ,\;x_{i+\left(m-1\right)\partial }\right\}$$

where m denotes the embedding dimension and ∂ denotes the time delay factor.

Then, the distances between two different vectors are defined as the maximum difference of their corresponding components as follows:

(2) $$d\left[X_{m}^{\partial }\left(i\right),\;X_{m}^{\partial }(j)\right]=\max\left\{\left|x_{i+k\partial }-\;x_{j+k\partial }\right|\;:0\leq k\leq m-1\right\}$$

where *i* and *j* are not equal.

The criterion for similarity is defined if the distance $d\left[X_{m}^{\partial }\left(i\right),\;X_{m}^{\partial }\left(j\right)\;\right]$ is less than a threshold parameter r. When the embedding dimension is *m* and *m* + 1 ($B_{i}^{m}$ and $B_{i}^{m+1}$1, respectively):

(3) $$B^{m}=\;\frac{1}{N-m\partial }\;\sum_{i=1}^{N-m\partial }B_{i}^{m},\;B^{m+1}=\;\frac{1}{N-m\partial }\;\sum_{i=1}^{N-m\partial }B_{i}^{m+1}$$

the SampEn can be finally defined:

(4) $$SampEn\;\left(x_{N},m,\;r,\;\partial \right)=\;-\ln\left[\frac{B^{m+1}}{B^{m}}\right]$$

In the present study, *r* was defined as 0.2^*^σ, where σ is the standard deviation of the original time series *x*_*N*_ (Stergiou, 2016).

Multiscale entropy (MSE) was used to quantify the level of regularity in HR and speed across multiple time scales, for each different training scenario. MSE integrates a coarse graining procedure to the SampEn algorithm to calculate the entropy value at each time scale, affording insight into the point-to-point fluctuations over a range of time scales, as follows:

(5) $$y_{j}^{\tau }=\;1/\tau \sum_{i=\left(j-1\right)\tau +1}^{j\tau }x_{N},\;1\leq y_{j}\leq N/\tau$$

where τ is the timescale of interest, *y*_*j*_ is a data point in the constructed time series, *x*_*N*_ is a data point in the original time series and *N* is the length of the original time series.

The reconstruction of the embedding dimension and definition of the time lag (using average mutual information) were performed for the time series individually (Goldberger Ary et al., 2000; Costa et al., 2002, 2005). The area under the multiscale entropy curves were also calculated to provide an insight of the integrated complexity of the variables over all time scales, and was defined as the complexity index (Busa and van Emmerik, 2016).

### Data Analysis

Speed and HR SampEn of the original time series and its surrogates were compared to verify whether the variability found in the data was not only a product of random noise (Stergiou, 2016). Thus, the data normality was tested using the Shapiro-Wilk test and, as the hypothesis of data coming from a normal distribution was rejected, the Mann–Whitney non-parametric test was applied (*P* < 0.05). A one-way repeated measures ANOVA was performed to compare the effect of different running routes in the entropy variables (*P* < 0.05). To determine the differences in the cognitive performance pre and post running trials, a one-way ANCOVA was performed (*P* < 0.05). The *post-hoc* was performed using magnitude-based inferences and precision of estimation (Batterham and Hopkins, 2006; Wilkinson and Winter, 2018). The speed and HR SampEn, as well as the complexity index across running routes with different degrees of familiarity (usual and unusual routes) and sensorimotor stimuli (standard 400-m track) were compared through post-only crossover spreadsheet (Hopkins, 2017). To realize the possible decrease/increase effects of running routes on athletes' cognitive measurements, data were analyzed using a specific spreadsheet for pre-post crossover trial (Hopkins, 2017). Differences in group means were expressed in raw units with 90% confidence limits. Smallest worthwhile differences were assessed using the standardized units multiplied by 0.2. Uncertainty in the true effects of the conditions were evaluated through non-clinical magnitude-based inferences. Magnitudes of clear effects were considered using the following scale: >5%, unclear; 25 to 75%, possibly; 75 to 95%, likely; 95 to 99%, very likely; >99%, most likely (Hopkins et al., 2009). Standardized (Cohen) mean differences, and respective 90% confidence intervals were also computed as magnitude of observed effects sizes (Batterham and Hopkins, 2006; Hopkins et al., 2009; Wilkinson and Winter, 2018). Thresholds for effect size statistics were: <0.2, trivial; 0.6, small; 1.20, moderate; 2.0, large; and >2.0, very large (Hopkins et al., 2009). The analysis of the relative variability in the terrains covered by runners in their individual trials was performed by calculating the coefficient of variation (CV) in the altitude time series.

## Results

### Heart Rate and Speed Entropies

The mean SampEn of the usual, unusual and 400 m track surrogates for HR (0.021 ± 0.01; 0.015 ± 0.006 and 0.12 ± 0.08, respectively) and speed (0.25 ± 0.18; 0.18 ± 0.12 and 0.85 ± 0.38, respectively) were significantly higher (*P* < 0.05) than the SampEn of the original time series (mean and standard deviation, SD, are described in Table 1), indicating that the variability contents of HR and speed are meaningful from the non-linear perspective.

**Table 1:** Descriptive and practical inferences of the speed and heart rate (HR) entropy measures across running routes with different degrees of familiarity (usual and unusual routes) and sensorimotor stimuli (standard 400-m track).

**Variables (a.u.)**	**Usual (mean ± SD)**	**Unusual (mean ± SD)**	**400 m (mean ± SD)**	**Group comparison outcomes as:**		
				**Mean changes (raw;** **±** **90%CL)**		
				**% Chances (decrease/trivial/increase)**		
				**Practical inferences**		
				**Usual vs. Unusual**	**Usual vs. 400 m**	**Unusual vs. 400 m**
Speed SampEn	0.22 ± 0.18	0.16 ± 0.11	0.81 ± 0.42	−0.36; ± 0.6	2.11; ± 0.60	2.54; ± 0.74
				63/24/13	0/0/100	0/0/100
				possibly –ive	most likely +ive	most likely +ive
HR SampEn	0.004 ± 0.003	0.004 ± 0.002	0.022 ± 0.026	0.08; ± 0.60	2.33; ± 0.84	2.2; ± 0.70
				21/43/36	0/0/100	0/0/100
				unclear	most likely +ive	most likely +ive
HR complexity index	0.56 ± 0.31	0.66 ± 0.33	3.10 ± 1.29	0.29; ±0.74	3.08; ± 0.88	2.80; ± 0.80
				13/29/58	0/0/100	0/0/100
				unclear	most likely +ive	most likely +ive

The ANOVA showed an effect of the running routes in the speed SampEn [*F*_(2, 16)_ = 16.99, *P* < 0.001, partial eta squared = 0.321]. Differences were also identified for the HR complexity index [*F*_(2, 16)_ = 6.73, *P* = 0.008, partial eta squared = 0.457]. The practical inferences for the *post-hoc* multicomparison among all entropy variables are described in Table 1. SampEn for speed showed to be possibly lower for unusual routes when compared to usual routes (moderate effect). The complexity index and SampEn for HR presented unclear mean changes, although the complexity index showed an almost 60% chance of increase for unusual routes when compared to usual routes. Overall, the entropy measures for the 400 m track presented higher values when compared to usual and unusual routes (large to very large effects), so it was the training scenario that demanded lower regularity in a single timescale for HR and speed, and with a multiple range of time scales for HR.

### Terrain Altitude

**Figure 1:**
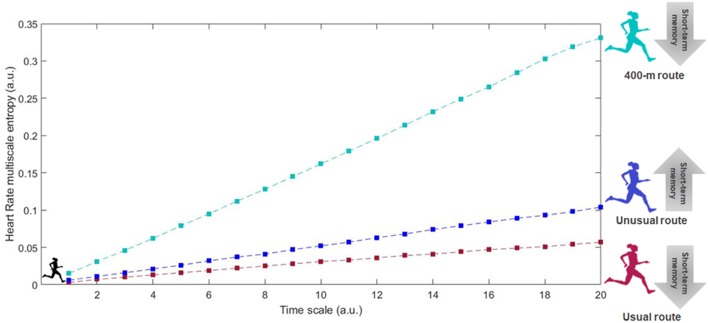
Representative case of the physiological factors in the multiscale entropy dynamics associated to training routes with different degrees of familiarity and sensory-motor stimuli, and its effects on life health span in adults. The usual route would be the most practiced or the standard trail/road route covered in individuals regular training sessions. The unusual route was chosen as the trail/road route which had never been part of training routine before the study assessments. The 400-m track was chosen to represent the sensory-motor stimuli of a flat, repetitive, thus, monotonous environment of practice.

The coefficient of variation in the altitudes covered by the runners in the usual and unusual training scenarios was very low. The usual routes of runners presented a CV of 3.3% ± 1.4% in the terrain altitude, while the unusual routes showed a CV of 4.6% ± 2.7%. As expected, the 400-m track presented a negligible CV of 0.2%. Figure 1 depicts a summary of the mechanic and physiological regularity, as well as short-term memory outcomes the different training scenarios.

### Cognitive Outcomes

Table 2 describes the results of the cognitive test pre and post all training scenarios. After training in usual routes, runners had a likely decrease in the levels achieved in the test (with 85% of chances), compared to the performance after training in unusual routes. Although it was not consistent, there is a 66% chance of decrease of the number of correct attempts after running in a 400-m track. There were no consistent differences in the number of fails in the cognitive test after running in the 400-m track and unusual route, but the results showed chances between 69 and 73% of increase, respectively. The average time to perform the test was not different between usual and unusual routes compared to the 400-m track, but most likely decreased after training in usual, compared to unusual, routes.

**Table 2:** Descriptive and practical inferences of the Backward digit-span performance outcomes pre and post running in routes with different degrees of familiarity (usual and unusual routes) and sensorimotor stimuli (standard 400 m track).

**Cognitive Outcomes**	**Usual route(mean** **±** **SD)**			**Unusual route(mean** **±** **SD)**			**400 m track(mean** **±** **SD)**			**Group comparison outcomes as:**		
										**Mean changes (raw;** **±** **90%CL)**		
										**% Chances (decrease/trivial/increase)**		
										**Practical inferences**		
										**Effect size (Standardized Cohen;** **±90%CL)**		
	Pre	Post	Δ	Pre	Post	Δ	Pre	Post	Δ	Usual vs. Unusual	Usual vs. 400 m	Unusual vs. 400 m
Level (*n*)	5.4 ± 0.7	6.1 ± 1.1	0.8 ± 0.9	6.4 ± 1.7	6.5 ± 1.2	0.1 ± 1.1	6.3 ± 1.1	6.4 ± 2.2	0.3 ± 2.1	−0.6; ± 0.6	−0.5; ± 1.6	0.2; ± 1.7
										85/14/1	62/16/22	32/24/45
										likely –ive	unclear	unclear
										−0.44; ±0.41	−0.43; ±1.46	0.11; ±1.15
Correct (*n*)	7.6 ± 1.2	9.0 ± 1.9	1.4 ± 1.6	8.8 ± 2.7	9.6 ± 2.5	0.9 ± 2	9.7 ± 1.9	9.6 ± 2.8	0.4 ± 2.2	−0.5; ± 1.8	−0.9; ± 2.5	−0.4; ± 1.7
										54/29/18	66/16/17	49/33/18
										unclear	unclear	unclear
										−0.23; ±0.8	−0.49; ±1.29	−0.18; ±0.72
Failed (*n*)	3.1 ± 0.8	3.3 ± 0.7	0.1 ± 0.8	3.0 ± 1.2	4.1 ± 3.6	1.1 ± 4.1	3.1 ± 0.9	3.9 ± 1.2	0.7 ± 2.1	1.0; ± 2.9	0.6; ± 1.3	−0.4; ± 3.7
										23/8/69	14/13/73	54/8/38
										unclear	unclear	unclear
										0.95; ±2.79	0.67; ±1.42	−0.38; ±3.42
Average time (*s*)	4.8 ± 1.3	7.1 ± 4.5	2.3 ± 4.4	6.3 ± 3.7	5.2 ± 1.0	−1.2 ± 4.5	5.0 ± 1.3	5.5 ± 1.6	0.4 ± 1.1	−3.5; ± 3.9	−1.9; ± 2.8	1.6; ± 3.3
										90/5/4	86/5/9	12/16/72
										likely –ive	unclear	unclear
										−1.18; ±1.32	−1.45; ±2.1	0.53; ±1.09

## Discussion

The present study aimed to identify the effects of habituation in the environments and contexts of outdoor running through the degree of regularity in speed, HR, and short-term memory performance in a group of runners. Habituation refers to familiarity over the environment of practice related to individuals' usual training route, a certain degree of unpredictability in the environment of practice at an unusual training route, and, finally, the monotony over a practice at the standard 400 m track. Although mechanical, physiological and cognitive aspects of physical exercise while interacting with surroundings rich with inherently different sensory-motor stimuli have already been reported in literature, it has never been analyzed as to how it is affected by time structure in training tasks, here represented by habituation levels over practice environments.

From a dynamical systems perspective, it was hypothesized that usual routes would demand decreased complex fluctuation patterns in major biological responses when compared to unusual routes. The HR complexity index presented a tendency to be higher in unusual, when compared to usual, routes. Aerobic fitness and HR variability are closely related, as previously reported in literature, although most of the studies available have used linear methods to analyse HR signal magnitude (Plews et al., 2012; Nakamura et al., 2015). By stimulating some degree of unpredictability in training, there is a solicitation of the mechanism underlying running endurance through the increase of parasympathetic activity (Da Silva et al., 2014), which enhances cardiorespiratory fitness (Sumi et al., 2006). The decrease in HR complexity across time and the further impacts on health has been an issue of interest in literature for over two decades (De Meersman, 1993; Costa et al., 2002) and can still be considered a hot topic in recent times (Togo and Takahashi, 2009). Consequently, health status is closely related to the degree of complex variability in system function, specially by its susceptibility to its beneficial and adaptive aspects. This premise lays on the loss of complexity hypothesis of Lipsitz (2002), which states that a path to frailty is identified by a certain amount of loss of variability in the fundamental outcomes that reflect aging or biological function over time, leading to the emergence of an injury or disease.

The present entropy results indicate that the contexts of practice may also influence the level of interactions in the complex network of physiological pathways. For example, the analysis of SampEn and complexity index provides a wider idea about the extent of the alterations in the HR and speed variability. While the first measure indicates, in a single time scale, that different sensory-motor stimuli increases biological signals unpredictability, the second summarizes the interaction of multiple scales through time, aligning to the multiscale complexity of the human body as a biological system (Costa et al., 2002).

In this sense, it was hypothesized that the usual route would yield lower single and multiscale regularity in speed and HR than the 400 m track race. The variation in the altitude terrain along the task elicited mechanical adjustments; running on uneven ground demands different timing for the muscle activation of lower limbs (Oliveira et al., 2016) and modifies kinematic gait patterns (Muller and Blickhan, 2010; Sinclair et al., 2013), so runners are able to alter step length and frequency during the task (Schubert et al., 2014). In fact, these are strategies that allow dynamic stability to cope with the terrain uncertainties while navigating the changing running surface (Muller and Blickhan, 2010; Larsen et al., 2016), thus, adapting to the modifications of the environment. It was found, though, that SampEn for speed, and consequently HR, was the highest for the standard 400 m track race when compared to unusual and usual routes. These findings may be related to the boring and monotonous quality of continuous running in a 400 m track for prolonged time. Monotony is related to the degree of predictability and affects familiarity and habituation over a task, as running in flat, round and repetitive terrain for a long period of time (Scerbo, 1998; Thiffault and Bergeron, 2003). In addition, the task-capability interface model suggests that the control of speed during a task can be influenced by the cognitive workload being experienced, as an attempt to increase the arousal levels (Fuller, 2005). Therefore, speed variability during prolonged sustained activity is increased in order to feel less sleepiness, fatigue and keep better vigilance over the task (Ma et al., 2018). The participants of this study reported, indeed, that using the 400 m track for middle- and long-distance running practice or training is rare, so it is an environment less visited. Additionally, when the amateur runners experienced a training session at the unusual route, picked as one never used to practice before the assessments, although it was not clear that it stimulated lower speed regularity, there was a tendency of the HR complexity index to be higher.

The present results align with other studies that explored the temporal capacity of structured and organized training in balance flexibility and stability so the benefits of variability in system function would occur (Moras et al., 2018). However, even though training in new terrains outdoors is challenging, less monotonous than the 400-m track, and mechano-physiologically varied, it is also under the training principle of adaptation. The training environment, i.e., routes defined for training sessions, should also be altered at a certain point in time to increase the demands of the body dynamic interactions. That would lead to higher biological entropy, thus enhancing body adaptive capacity to the training effects over time (Busa and van Emmerik, 2016).

Another component essential to integrate and interpret sensorimotor information of everyday life is cognition, which is also affected by age (Reuter-Lorenz and Park, 2010). Physical activity is reported to affect brain plasticity and positively influence cognition and well-being (Gutchess, 2014; Mandolesi et al., 2018), while variability holds it all together (Woollett and Maguire, 2011). Hereupon, the effect of different environments of practice regarding to route familiarity and sensory motor stimuli in short-term memory was also verified. The participants showed higher overall performance on the short-term memory test after practicing in the unusual, when compared to the usual, route. Short-term memory is specialized for the temporary storage of information within particular informational domains. It is also related to mechanisms of the attentional control in reactivating memory traces and inhibiting irrelevant information (St Clair-Thompson, 2010). Although unclear, results of the overall performance showed a tendency to worsen after practice in the 400 m track when compared to usual routes. There was also a tendency of decreasing the number of correct sequences after practice in the 400 m track, compared to the unusual route. Repetitive or absent stimulation is reported to generate cognitive underload (Larue, 2010), which could be related to the familiarity in the usual route or the monotony of prolonged running in a 400 m track. Low cognitive workload leads to hypervigilance due to a lack of desire to continue performing the task, and negatively affects the vigilance and alertness state, as well as attention (Sussman and Coplen, 2000). Even though the runners varied speed as a strategy to suppress monotony in the continuous running at the 400 m track, it may not have been successful to avoid cognitive underload. The unclear results for the cognitive test, though, might have been limited by the sample size. We understand that the profile of participants for the sample should approximate the model which integrates the effects of aging, exercise and health in master athletes (Lazarus and Harridge, 2007), however, it is challenging to find large groups of runners engaged in amateur competitions who are homogeneous in terms of involvement in organized training.

The present study described how runners regulate mechanical and physiological responses during training practice under courses that differ in its level of familiarity and sensorimotor stimuli. Higher entropies were found for the 400 m track race, compared to usual and unusual routes. Usual routes increased speed SampEn, but decreased HR complexity index when compared to unusual. Runners showed higher overall short-term memory performance after unusual routes, when compared to usual routes. Although runners might be able to vary speed, thus affording higher entropy levels to HR responses, at cognitive level this is not very effective. Thus, by approaching this issue with non-linear analysis, it was possible to identify evidences on the importance of manipulating basic training constraints to increase the adaptive demands and avoid routines that can preclude such adaptations. New research lines should consider different contextual variations to investigate issues related to training adaptability in runners.

## Data Availability

All datasets generated for this study are included in the manuscript and/or the supplementary files.

## Ethics Statement

The study protocol was conformed to the recommendations of the Declaration of Helsinki, and was approved and followed the guidelines stated by the local Institutional Research Ethics Committee.

## Author Contributions

JS: conceptualization. JE, NM, BG, and CA: methodology. JE and BG formal analysis. JE and JS: writing—original draft preparation. JE, BG, NM, CA, JC-G, and JS: writing—review and editing.

## Contribution to the Field

General population physical activity levels are a global concern but its regulation seems tricky in modern society. The gap in the practical and academic approach might be in not considering how specific behaviors from specific socio-environmental agents determine motivation to change habits and maintain engagement in physical activity. Humans tend to repeat and stabilize decisions and behaviors in daily life, and choices of physical activity practice and training contexts are constantly under the influence of habituation, leading to monotony. The lack of variability in daily life might impair an important link in a healthy body function: action and cognition. When one's perception capabilities are challenged by the surroundings, there is an enhance of body capacity in coordinating activities, as well as increased levels of awareness. Thus, by considering the environment-individual relationship in physical activity as a complex system, the current study brings novel contributions on how habituation of training contexts affects runners physiological and cognitive adaptations. Although performers find a way to increase physiological variability in highly monotonous and habitual tasks, it is still not effective in increasing arousal, thus favoring cognitive impairment, even in acute level.

### Conflict of Interest Statement

The authors declare that the research was conducted in the absence of any commercial or financial relationships that could be construed as a potential conflict of interest.
